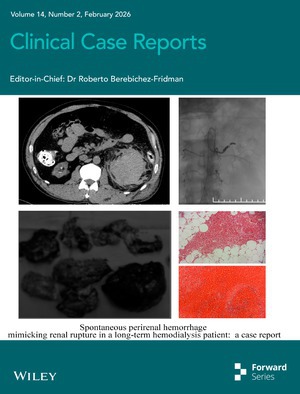# Cover Image

**DOI:** 10.1002/ccr3.72210

**Published:** 2026-03-05

**Authors:** Zhen Wang, Bo Yang, Yi Zhang, Xiaoqiang Li, Bin Wang, Jinghan Chen

## Abstract

The cover image is based on the article *Spontaneous Perirenal Hemorrhage Mimicking Renal Rupture in a Long‐Term Hemodialysis Patient: A Case Report* by Zhen Wang et al., https://doi.org/10.1002/ccr3.72008.